# Epidermal growth factor signalling pathway in endochondral ossification: an evidence-based narrative review

**DOI:** 10.1080/07853890.2021.2015798

**Published:** 2021-12-27

**Authors:** L. Mangiavini, G. M. Peretti, B. Canciani, N. Maffulli

**Affiliations:** a IRCCS Istituto Ortopedico Galeazzi, Milan, Italy; b Department of Biomedical Sciences for Health, Università Degli Studi di Milano, Milan, Italy; c Department of Medicine, Surgery and Dentistry, University of Salerno, Baronissi, SA, Italy; d Barts and the London School of Medicine and Dentistry, Centre for Sports and Exercise Medicine, Queen Mary University of London, London, UK; e School of Pharmacy and Bioengineering, Keele University Faculty of Medicine, Stoke on Trent, UK

**Keywords:** Epidermal growth factor, bone development, endochondral ossification

## Abstract

During endochondral bone development, a complex process that leads to the formation of the majority of skeletal elements, mesenchymal cells condense, differentiating into chondrocytes and producing the foetal growth plate. Chondrocytes progressively hypertrophy, induce angiogenesis and are then gradually replaced by bone. Epidermal Growth Factor (EGF), one of many growth factors, is the prototype of the EGF-ligand family, which comprises several proteins involved in cell proliferation, migration and survival. In bone, EGF pathway signalling finely tunes the first steps of chondrogenesis by maintaining mesenchymal cells in an undifferentiated stage, and by promoting hypertrophic cartilage replacement. Moreover, EGF signalling modulates bone homeostasis by stimulating osteoblast and osteoclast proliferation, and by regulating osteoblast differentiation under specific spatial and temporal conditions. This evidence-based narrative review describes the EGF pathway in bone metabolism and endochondral bone development. This comprehensive description may be useful in light of possible clinical applications in orthopaedic practice. A deeper knowledge of the role of EGF in bone may be useful in musculoskeletal conditions which may benefit from the modulation of this signalling pathway.

**Key messages**
1. The EGF pathway is involved in bone metabolism.
2. EGF signalling is essential in the very early stages of limb development by maintaining cells in an undifferentiated stage.
3. EGF pathway positively regulates chondrocyte proliferation, negatively modulates hypertrophy, and favours cartilage replacement by bone.
4. EGF and EGF-like proteins finely tune the proliferation and differentiation of bone tissue cells, and they also regulate the initial phases of endochondral ossification.

## Introduction

### Endochondral bone development

Bones form through two complex processes: intramembranous or endochondral ossification. During the former, mesenchymal cells directly differentiate in osteoblasts by activating the RUNX-2 pathway. This process occurs in most of the calvarial bones and in the clavicle [1]. Endochondral ossification is more complex, and it involves an initial cartilage anlage, which is then replaced by bone [1]. Mesenchymal progenitors first condensate and then start differentiating into chondrocytes. These latter cells pile up in columns, exit the cell cycle, and secrete an osteogenic matrix and pro-angiogenic factors, such as Vascular Endothelial Growth Factor (VEGF) [2,3]. Subsequently, perichondrial cells surrounding the primary cartilage anlage invade the template together with blood vessels, and they differentiate into osteoblasts, forming the primary ossification centre. Subsequently, chondrocytes form the growth plate at both ends of the primary ossification centre [4].

**Figure 1.**
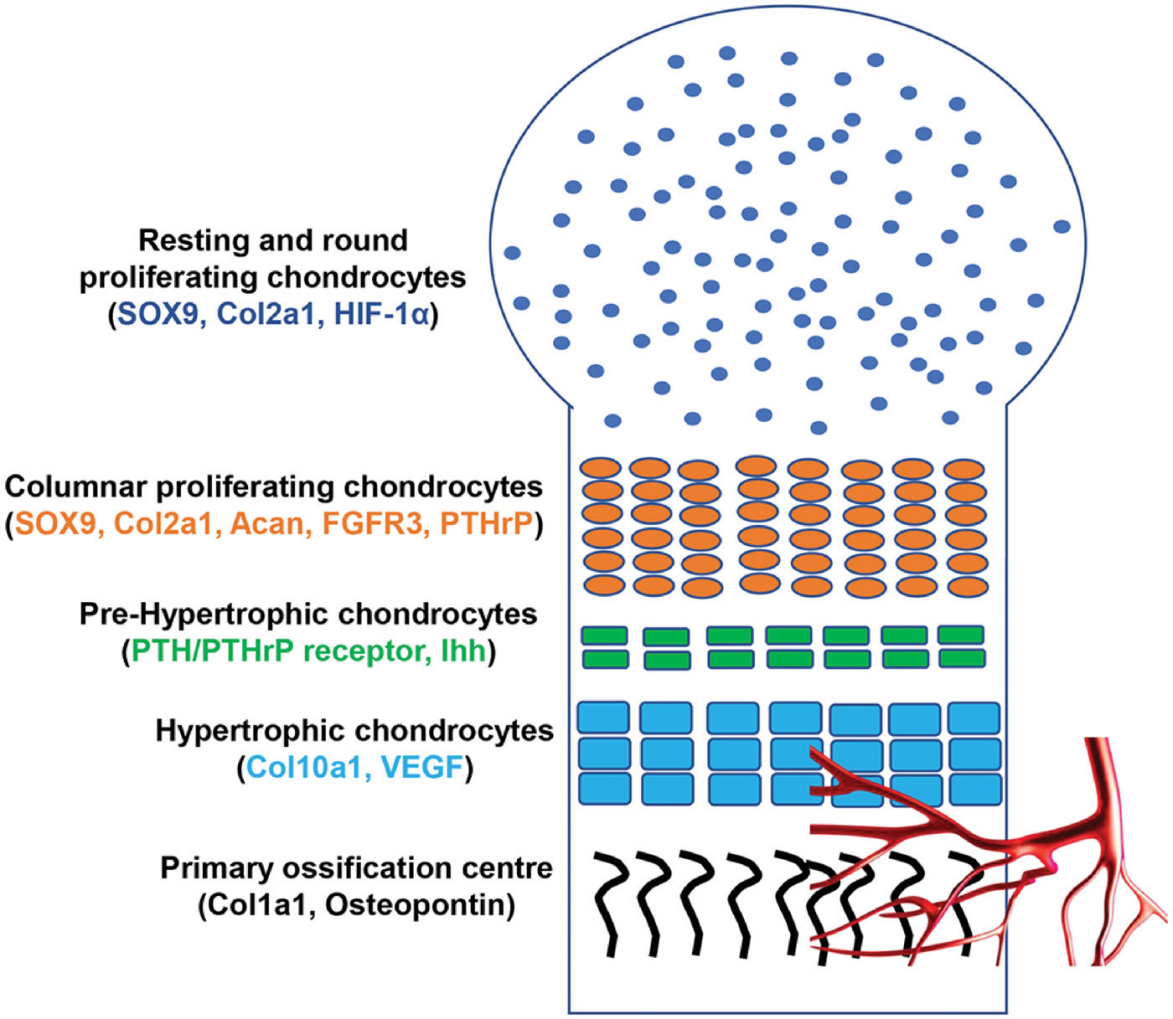
Schematic representation of the formation of the growth plate during endochondral bone development. In the resting zone, chondrocytes have a round shape and they mainly express SOX9, Col2a1 and HIF-1α. In the proliferating layer, chondrocytes express SOX9, Acan, Col2a1, PTHrP, FGFR3; they assume a cuboidal shape, progressively pile up in columns. Columnar chondrocytes stop proliferating and they firstly become pre-hypertrophic cells, which express PTHrP receptor and Ihh. These cells terminally differentiate into hypertrophic cells, which express Col10a1 and VEGF; thus promoting angiogenesis. Lastly, hypertrophic cells are progressively replaced by osteoblasts expressing Col1a1 and osteopontin.

The growth plate is composed of different layers, each representing a distinct stage of cell differentiation (Figure 1). In the resting zone, chondrocytes have a round shape, are still in an undifferentiated phase and divide asymmetrically: some of them remain as “stem cells,” and the others differentiate into proliferative chondrocytes, which form the proliferating layer. In this layer, chondrocytes assume a cuboidal shape, they floridly proliferate, and progressively pile up in columns, forming the proliferating columnar layer. Gradually, columnar chondrocytes exit the cell cycle, stop proliferating and they first become pre-hypertrophic cells, which then terminally differentiate into hypertrophic chondrocytes [4].

Most of these latter cells undergo apoptosis and are resorbed by osteoclasts. Conversely, some of these cells directly transdifferentiate into osteoblasts [5]. Thus, both stem cells from the resting zone and hypertrophic chondrocytes maintain their osteogenic potential [5]. During this process, the cartilage template progressively enlarges, allowing for bone growth.

In the growth plate, cell gene expression varies depending on the layer, and this influences cell fate and differentiation. Mesenchymal progenitors express Sox-9, a master transcription factor for chondrocytes differentiation [6–8]. Upon Sox-9 pathway activation, chondrocytes in the proliferating zone largely express type II collagen alpha 1 (Col2a1), the main component of the cartilaginous matrix, aggrecan (Acan), fibroblast growth factor receptor 3 (FGFR3) and Parathyroid hormone-related peptide (PTHrP). Conversely, pre-hypertrophic cells express PTH/PTHrP receptor and Indian hedgehog (Ihh) mRNA, and hypertrophic chondrocytes are characterised by an abundant expression of type X collagen alpha 1 (Col10a1); thus, they contribute to the secretion of osteogenic matrix [4].

The foetal growth plate is highly hypoxic. Thus, the activation of the hypoxia-inducible factor pathway is crucial for chondrocyte survival and differentiation [9–11]. Moreover, this pathway also initiates blood invasion at the level of the primary ossification centre through the activation of the Vascular Endothelial Factor (VEGF) [12]. Many other growth factors play an important role in endochondral bone development, such as FGFR3 [13,14]. For example, gain-of-function mutations of this molecule cause achondroplasia, a genetic bone dysplasia [15].

### Epidermal growth factor (EGF) signalling pathway

Recently, the role of the Epidermal Growth Factor (EGF) in bone development has gained interest, as it is involved in multiple steps of this process [16]. EGF is a single-chain polypeptide consisting of 53 amino acids, with three intramolecular disulphide bonds, which are essential for its function [17,18]. EGF is the prototype of the EGF-ligand family which includes other EGF-like proteins, such as Heparin-Binding (HB-EGF), betacellulin (BTC), Transforming Growth Factor-α (TGF-α), Epigen (EPGN), amphiregulin (AREG), epiregulin (EREG) and neuregulins (NRG1-4) [19,20]. These molecules share a high affinity for the EGF receptor, a similar response in cells and a typical 35–40 amino acid sequence spaced by 6 conserved cysteines in this order: CX_7_CX_3–5_CX_10–12_CXCX_5_GXRC (C: cysteine, G: glycine, R: arginine, X: other amino acids). The six cysteines form the three disulphide bonds, which are the hallmark of the EGF-ligand family [21,22].

The EGF receptor (EGFR or ErbB1) has a typical transmembrane structure: an extracellular domain and a tyrosine kinase cytoplasmic domain. EGFR is activated either through an autocrine mechanism, where different stimuli lead to the synthesis of EGFR ligands or through an intra-cellular transactivation process [23].

**Figure 2.**
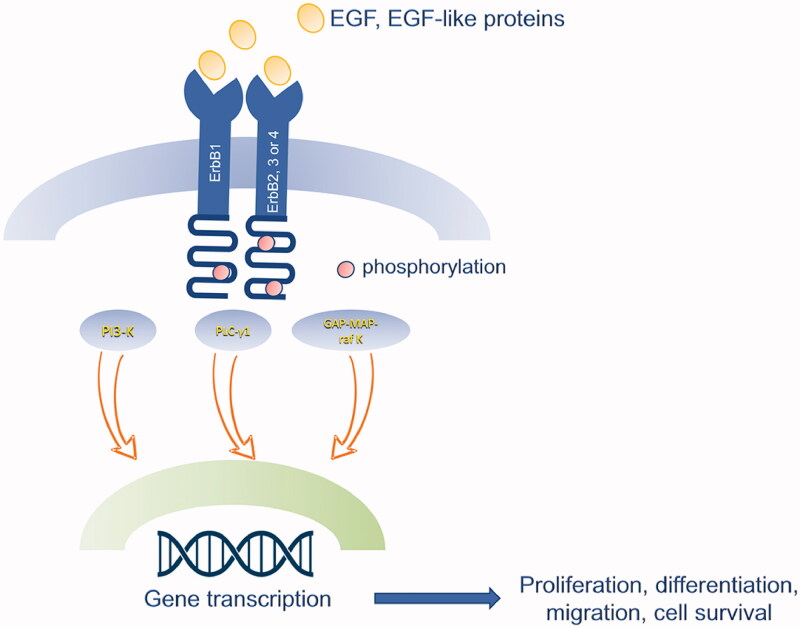
Schematic representation of EGF signalling pathway. The binding of EGF or EGF-like proteins induces oligomerization of EGFR (ErbB1) with ErbB2/c-neu, ErbB3, and ErbB4, and subsequent phosphorylation of the receptor, leading to the transduction of different pathways: PLC-γ1, PI-3 Kinase, GAP-MAP-*raf* kinase. These pathways are involved in several functions, such as cell proliferation, migration and survival.

The binding of EGF or EGF-like proteins induces oligomerization of EGFR with ErbB2/c-neu, ErbB3, and ErbB4, and subsequent phosphorylation of the receptor, leading to the transduction of different pathways: PLC-γ1, PI-3 Kinase, GAP-MAP-*raf* kinase. These pathways are involved in several functions, such as cell proliferation, migration and survival. PI-3 and MAP kinase transduction are also mainly involved in endothelial cells migration and vessel formation in several tissues; thus, they promote angiogenesis (Figure 2) [24,25].

EGFR ligands can be divided into three groups based on the affinity for the different subunits of the receptor. EGF, TGF-α and AREG can only activate ErbB1; NRG1-4 can bind ErbB3 and ErbB4; and finally HB-EGF, BTC and EREG activate both ErbB1 and ErbB4. ErbB2 does not directly bind any ligand but it can dimerise with all the other subunits [23].

The EGF signalling pathway is involved in several tissues at different stages of development. Indeed, EGF stimulates blastocyst formation and embryo implantation [22]. Postnatally, it is required for a proper formation of the gastrointestinal tract, for lactation and for regulation of body mass [24].

EGF also plays a central role in wound healing; exogenous application of EGF associated with several scaffolds represents a valid tool in wound healing [22]. In bone, the EGF signalling pathway has also been involved both in oncological and inflammatory pathologies, as briefly described in the next two sections.

#### EGF signalling pathway in bone tumours

EGF is implicated in cancer: its tyrosine kinase activity is responsible for tumour survival, growth and metastatization [26]. EGF favours epithelial-mesenchymal transition (EMT) and it maintains cancer stemness [27,28]. In bone, this pathway is involved both in primary malignant tumours and in the process of metastases formation. In osteosarcoma, EGF activates the MAPK/ERK and PI3K/Akt pathways, thus leading to cytoskeleton reorganisation, which in turn causes cell proliferation and migration [29,30]. Moreover, EGFR is expressed in osteosarcoma cells, it is associated with a worse prognosis, and it is related to local recurrences and metastatization [31–33]. EGFR activity is also linked to chemotherapy resistance and cell survival under stress conditions, suggesting that the inhibition of EGFR associated with antineoplastic drugs might exert a synergistic effect on tumour progression [31].

In osteolytic bone metastasis, tumour cells secrete osteoclastogenic factors (i.e. RANKL, interleukin-6, PTHrP), which activate osteoclasts. The release of growth factors by the resorbed bone further stimulates cancer cells, thus leading to a vicious cycle [34]. EGF signalling may further sustain this vicious cycle by downregulating the RANKL-antagonist Osteoprotegerin (OPG), ultimately increasing osteoclast activity [34,35].

#### EGF signalling pathway in osteoarthritis

EGFR activity is also present in the superficial layer of healthy articular cartilage, and it is greatly diminished in osteoarthritic samples [36–38]. This evidence point to the role of the EGF pathway in osteoarthritis (OA), as confirmed by several knockout murine models. Reduced EGFR activity in a murine model increased chondrocyte apoptosis and matrix degradation [39].

Moreover, mice lacking EGFR activity in chondrocytes (EGFR-Col2-Cre) display defective articular cartilage, and they quickly develop OA [40]. EGFR activity promotes chondrocyte proliferation of the superficial layer, it maintains joint lubrication and preserves the mechanical functions of cartilage [40]. Consistent with these findings, the deficiency of Mig6, an inhibitor of EGFR, led to an increased number and thickness of the superficial chondrocytes [38,41,42]. Conditional overexpression of EGFR enhances the pool of chondroprogenitor cells and it delays the process of osteoarthritic degeneration [43].

In contrast, EGFR activity was overexpressed in a subpopulation of OA patients, and *in vitro* cultures of chondrocytes with EGFR induction demonstrated a role of this pathway in the loss of cartilage homeostasis [44]. The intra-articular application of gefitinib, a specific EGFR inhibitor, ameliorated the OA phenotype in a mouse model [44].

Thus, the EGF signalling pathway has a contradictory role in OA. It is possible that EGFR activity may promote joint degeneration only in a specific subpopulation of OA patients. A different spatial and temporal activation of this pathway may also explain these conflicting results.

These findings open the road for novel therapeutic strategies targeting OA. Intra-articular injections of stabilised TGF-α in OA knees slows down the process of joint degeneration by reducing the catabolic activity, subchondral bone sclerosis and synovitis [43]. On the other hand, gefitinib-mediated EGFR inactivation may improve cartilage homeostasis in a specific subpopulation of OA patients.

EGF is therefore linked to several physiological and pathological bone conditions. In this review, we focussed on the EGF signalling pathway in the first steps of bone formation. We analyse the expression and function of EGF in bone homeostasis, and particularly in the endochondral development of bone.

## Materials and methods

We used the string EGF AND endochondral ossification or EGF AND osteogenesis to search the Pubmed and Web of Science databases to identify articles on the role of this growth factor in bone development. We divided these articles based on topics: role of EGF in cell proliferation or differentiation of bone tissue, the role of EGF in endochondral ossification.

## Results

**Figure 3.**
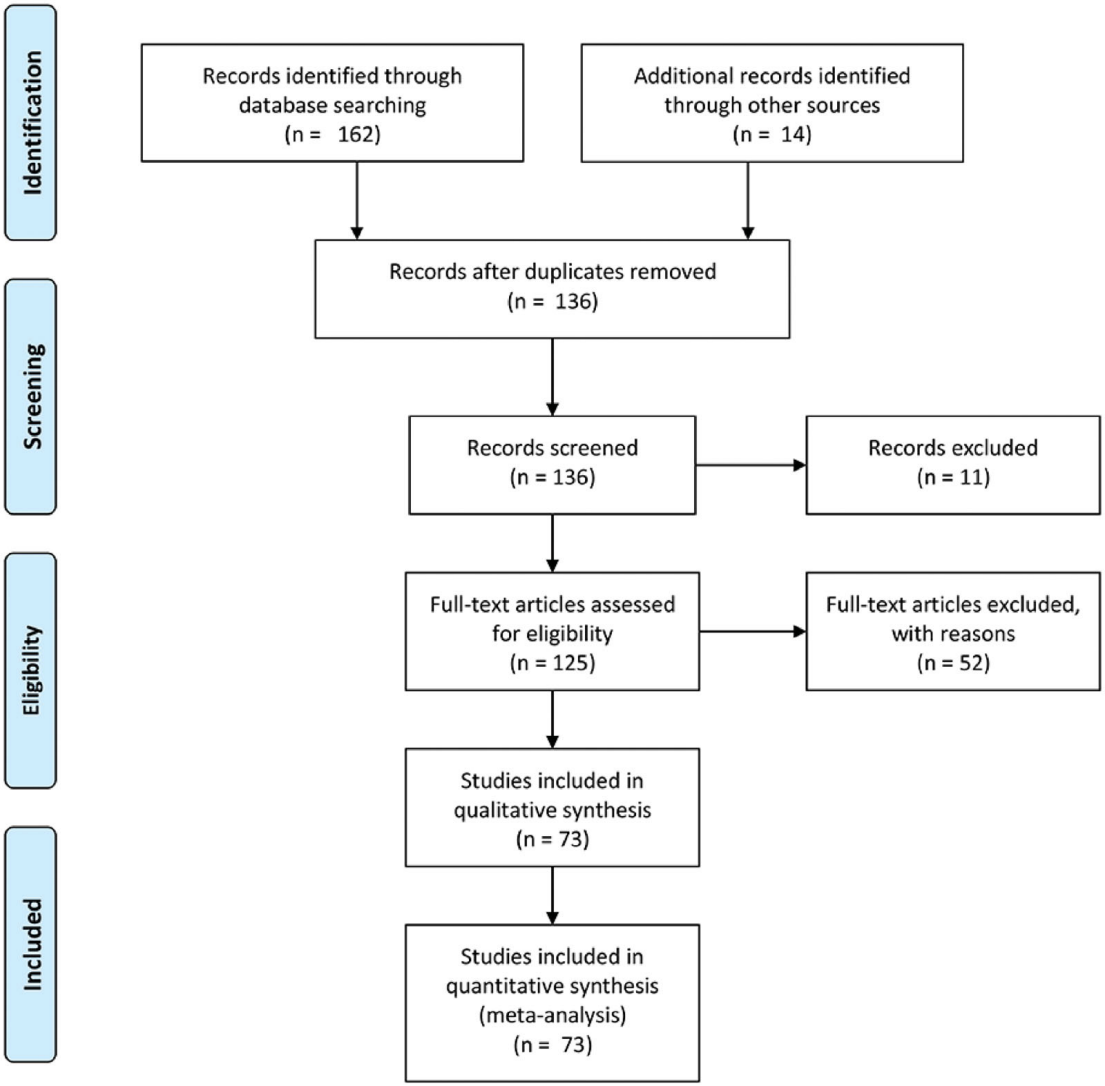
PRISMA flow diagram of the present systematic review.

We retrieved 162 articles: 73 manuscripts were included, 11 articles were excluded for the absence of full text, 52 articles were excluded because they studied unrelated topics. The selected manuscripts were divided into two main groups: projects on the role of EGF in proliferation and/or osteogenic differentiation (*n* = 46), and articles on the role of EGF in endochondral ossification (*n* = 13). Studies were mainly focused on the role of EGF in proliferation and osteogenic differentiation *in vitro* (*n* = 43). Only a few articles specifically analysed the EGF signalling pathway in endochondral ossification (*n* = 13). We identified additional 14 investigations from the reference lists of those articles to better understand the function of EGF in this developmental process (Figure 3). Based on the analysis of the articles identified, we describe the EGF signalling pathway in bone and in endochondral ossification (Tables 1 and 2).

**Table 1.** EGF in bone metabolism database.

Authors	Year	Type of study	Results
Ng, K.W., et al.	1983	*In vitro*, rat osteosarcoma cells	EGF promotes cell proliferation
Ng, K.W., et al.	1983	*In vitro*, rat osteosarcoma cells	EGFR is expressed in osteogenic cells
Takahashi, N., et al.	1986	*In vitro*, human marrow cultures	TGF-α stimulates osteoclast formation
Ibbotson, K.J., et al.	1986	*In vitro*, rat long bones and neonatal mouse calvariae	TGF-α and EGF promote bone resorption
Nakayama, Y., et al.	1990	*In vitro*, mouse MC3T3-E1 cells	EGF stimulates proliferation and inhibits differentiation
Joos, U.E., et al.	1992	*In vitro*, rat mesenchymal and osteoblast cells	EGF promotes osteogenesis in combination with TGFβ
Satomura, K., et al.	1998	*In vitro*, human BMSCs	EGFR is expressed in osteoprogenitors cells
Beech, D., et al.	1998	*In vitro*, human soft tissue sarcoma cells	EGF promotes cell proliferation and mitogenesis
Yarram, S.J., et al.	2004	*In vitro*, human osteoblast cell line MG63	EGF and calcitriol promote osteogenic differentiation
Lin, H.T., et al.	2005	*In vitro*, human BMSCs	EGF promotes proliferation
Qin, L., et al.	2005	*In vitro*, rat osteosarcoma cell line	AREG is expressed in osteoblasts and it is regulated by PTH
Tamama K., et al.	2006	*In vitro*, human and rat BMSCs	EGF promotes proliferation of human BMSCs
Elabd, C., et al	2007	*In vitro*, human ADSCs	EGF promotes osteogenic differentiation
Ozaki Y., et al.	2007	*In vitro*, rabbit and human MSCs	EGF and HB-EGF promote cell migration
Grasser, W.A., et al.	2007	*In vitro*, *in vivo*, human osteoblasts, mouse	EGF and IGF-1 promote osteogenic differentiation upon BMP-6 stimulus
Zhu, J., et al.	2007	*In vitro*, mouse osteoblastic cell line	EGF-like ligands stimulate osteoclastogenesis by acting on osteoblastic cells
Cheon S.J., et al.	2008	*In vitro*, human ADSCs	EGF and nsulin-transferrin-selenium (ITS) promote proliferation
McCarty, R.C., et al.	2009	*In vitro*, *in vivo*, ovine BMSCs, mouse	TGF-α promotes proliferation
Marcantonio, N.A., et al.	2009	*In vitro*, human BMSCs	Tethered EGF stimulates osteogenic differentiation
Platt, M.O., et al.	2009	*In vitro*, human BMSCs	Tethered EGF induces osteogenesis
Solmesky, L., et al.	2010	*In vitro*, human BMSCs	EGF and bFGF stimulate cell proliferation and migration
Laflamme, C., et al.	2010	*In vitro*, osteoblast-like cell line from human osteosarcoma	EGF has a synergistic effect with BMPs on cell proliferation
Tamama, K., et al.	2010	Review	Role of EGF In MSC proliferation and differentiation
Chieregato, K., et al.	2011	*In vitro*, human ADSCs	Combination of EGF, bFGF and PRP supports cell expansion
Zhu, J., et al.	2011	*In vitro*, mouse osteoblastic cell line	EGF-ligands suppress osteoblast differentiation and EGFR signalling downregulates Rux-2 and Osterix
Zhu, J., et al.	2012	*In vitro*, human and rat BMSCs	AREG stimulates mesenchymal cell migration towards PTH-stimulated osteoblasts
Nickerson, N.K., et al.	2012	*In vitro*, *in vivo*, breast and osteoblast cancer cell lines, mouse	Inhibition of EGFR signalling decreases tumour growth and metastatization
Yu, S., et al.	2013	*In vitro*, C2C12/Runx2^Dox^ cells	Runx2 induces osteogenesis by downregulating HB-EGF
Keeve, P.L., et al.	2013	*In vitro*, human periodontal and palate cells (pdSCs and paldSCs)	EGF promotes cell migration during osteogenesis
Hu, F., et al.	2013	*In vitro*, rat ADSCs	Low concentrations of EGF and bFGF limit osteogenic differentiation
Lim, K.T., et al.	2013	*In vitro*, human ABMSC	Fluid shear stress enhances EGF expression during osteogenic differentiation
Liu, X., et al.	2013	*In vitro*, *in vivo*, HEK293, C2C12 and C3H10T1/2 cell lines, mouse foetal limbs, mouse	EGF enhances BMP-9 induced osteogenesis of MSCs
Felthaus, O., et al.	2014	*In vitro*, dental follicle cells (DFCs)	EGF does not induce osteogenic differentiation
Yang, M., et al.	2014	*In vitro*, MC3T3-E1 cell line, mouse BMSCs and osteoblasts	miR-96 promotes osteogenic differentiation by inhibiting HB-EGF
Lee, H.L., et al.	2014	*In vitro*, C2C12 mouse cell line	Smurf1 mediates the inhibitory effect of EGF on BMP2-induced osteoblast differentiation
Tanaka, U., et al.	2015	*In vitro*, multipotent clonal human periodontal cell line	Spry2 combined with bFGF and EGF stimulation reduced cell migration and proliferation
Boonanantanasarn, K., et al.	2015	*In vitro*, HEK293T and C2C12 cell lines	EGF/Smurf1 inhibits Wnt/b catenin induced osteogenic differentiation
Del Angel-Mosqueda, C., et al.	2015	*In vitro*, human dental pulp stem cells (DPSCs)	EGF promotes osteogenic differentiation
Lee, J.H., et al.	2015	*In vitro*, *in vivo*, human MSCs, rat calvarial defects	Combination of EGF and rhBMP-2 enhances bone formation
Lee, J.H., et al.	2015	*In vitro*, *in vivo*, human MSCs, rabbit tibial defects	Combination of EGF and rhBMP-2 enhances bone formation in an orthotopic model
Kuek V., et al.	2016	*In vitro*, primary murine osteoblasts	NPNT is expressed in osteoblasts and favours angiogenesis
Ai, G., et al.	2017	*In vitro*, human ADSCs	EGF promotes cell proliferation
Go, Y.Y., et al.	2017	*In vitro*, MG-63 cell line, human BMSCs	EGF negatively regulates osteogenic differentiation
Sun, Y., et al.	2018	Review	NPNT, a novel EGF-ligand, is involved in angiogenesis-osteogenesis coupling
Wang, J., et al.	2020	*In vitro*, murine-macrophage cell line RAW 264.7, human MSCS	Ca-P ceramics increases EGF expression during osteogenic differentiation
Zou, W., et al.	2020	*In vivo*, mouse	Modulation of BMP signalling together with short-term EGF receptor activation increase bone mass

**Table 2.** EGF and endochondral ossification database.

Authors	Year	Type of study	Results
Chenevix-Trench, G., et al.	1992	*In vivo*, human	Cleft palate associated with polymorphism in TGFA gene
Threadgill, D.W., et al.	1995	*In vivo*, mouse	EGFR null mice have prenatal and postnatal defects and growth retardation based on the genetic background
Miettinen, P.J., et al.	1995	*In vivo*, mouse	EGFR null mice have epithelial alterations and dysfunctions in several tissues and growth retardation
Monsonego E., et al.	1995	*In vitro*, a*via*n epiphyseal chondrocytes	EGF and GH increase cell proliferation
Huang, L., et al.	1996	*In vitro*, rat micromass culture	TGF-α prevents chondrocyte differentiation
Bonassar, L.J., et al.	1997	*In vitro*, rabbit	EGF interacts with IGF-1 in skeletal growth
Dealy, C.N., et al.	1998	*In vitro*, embryonic chick limb	Exogenous TGF-a and EGF inhibit chondrogenesis and myogenesis of limb mesenchyme
Miettinen, P.J., et al.	1999	*In vivo*, mouse	EGFR null mice have impaired craniofacial development
Yoon, Y.M., et al.	2000	*In vitro*, micromass culture of chick limb bud	EGF negatively regulates chondrogenesis by modulating PKC, Erk-1, p38 MAPK
Chan, S.Y., et al.	2000	*In vivo*, mouse	Overexpression of human EGF impairs endochondral development
Sibilia, M., et al.	2003–2016	*In vivo*, mouse	Mice humanised for EGFS display growth retardation
Wang, K., et al.	2004	*In vivo*, mouse	EGFR-deficient mice have delayed primary endochondral ossification
Schneider, M.R., et al.	2005	*In vivo*, mouse	BTC overexpressing mice display growth retardation and reduced bone dimensions
Fisher, M.C., et al.	2007	*In vivo*, mouse	Inhibition of EGF signalling causes delayed endochondral ossification and impaired chondrocyte and osteoblast proliferation
Schneider, M.R., et al.	2009	*In vivo*, mouse	BTC overexpressing mice display increased BMD and bone cortical mass
Genetos, D.C., et al.	2010	*In vitro*, human MSCs	BTC promotes proliferation but it inhibits differentiation upon HIF-1α regulation
Zhang, X., et al.	2011	*In vivo*, mouse	EGFR conditional knockout in pre-osteoblasts causes reduced trabecular and cortical volume
Usmani S.E., et al.	2012	*In vivo*, mouse	TGF-α knockout model shows impaired endochondral development ut to 10 weeks postnatally
Hall, K.C., et al.,	2013	*In vivo*, *in vitro*, mouse	ADAM17 conditional knockout in chondrocytes displays a significant expansion of hypertrophic chondrocytes
Saito K., et al.	2013	*In vivo*, mouse	TACE conditional knockout in chondrocytes delays hypertrophy through EGFR signalling
Pruvot B., et al.	2014	*In vivo*, zebrafish	Inhibition of EGF signalling impairs Meckel’s cartilage development
Chim, S.M., et al.	2015	*In vitro*, *in vivo*, mouse	EGFL7 regulates angiogenesis in bone microenvironment
Lin, Y.C., et al.	2015	*In vitro*, *in vivo*, mouse	Scube2 knockout impairs Ihh-dependent endochondral ossification
Wolf, C.J., et al.	2018	*In vitro*, organoids from human umbilical MSCs	EGF promotes proliferation during palate development
Li P., et al.	2019	*In vitro*, *in vivo*, mouse BMSCs, mouse	HB_EGF overexpression in osteoprogenitor cells causes chondrodysplasia, chondromas and sorter long bones
Fang, R., et al.	2020	*In vivo*, mouse	iRhoms 1 and 2 conditional knockout in chondrocytes impairs endochondral ossification
Lin, Y.C., et al.	2021	*In vitro*, *In vivo*, mouse, human	Scube3 loss of function causes growth disorders by impairing BMP signalling

### Role of EGF signalling pathway in cell proliferation and differentiation of bone tissue

EGF, EGF-like genes and EGF-R are abundantly expressed in bone tissue, where they play a crucial role in cell proliferation, differentiation and in the coupling osteogenesis-angiogenesis [17,45,46]. Genes encoding for EGF signalling proteins are present in mesenchymal cells, osteoblasts, osteoclasts and endothelial cells. For example, activation of this pathway stimulates periodontal cell proliferation and inhibits differentiation [47,48].

EGF stimulates the proliferation of osteoblast-like cells [47,49–52]. In rat osteosarcoma cells, EGF induces the expression of Egr-1 mRNA, thus increasing mitogenesis [53]. In addition, a combination of EGF and Bone Morphogenic Proteins, namely BMP-2 and −7, further stimulates cell proliferation in the early differentiation stage, while inhibiting late osteoblast differentiation [53]. EGF also promotes proliferation and migration of mesenchymal cells from different origins (bone marrow, adipose tissue, Human Alveolar Bone-Derived Mesenchymal Stem Cells, periodontium-derived stem cells) [54–64].

Furthermore, EGF-like proteins are involved in bone metabolism [65]; in particular, amphiregulin (AREG) is highly expressed in preosteoblasts, where it strongly promotes proliferation upon Parathyroid hormone (PTH) regulation [66]. The secretion of AREG from osteoblasts and osteocytes stimulates mesenchymal cell chemotaxis and recruitment, thus favouring the anabolic function of PTH in bone [67].

All the EGF-like proteins strongly suppress osteogenesis in different cell lines *in vitro* [68,69]; EGFR mediates this effect by activating Smurf1, an E3 ubiquitin ligase, which in turn inhibits Wnt/βcatenin osteogenic differentiation downregulating the master transcription factors of osteoblastogenesis, Runx2 and Osterix [69–75].

Conversely, the EGF signalling pathway has been also involved in osteoblast differentiation [76–81]; in MG63 immature osteoblasts, the combination of calcitriol and EGF leads to an increase in alkaline phosphatase and osteocalcin protein expression in a dose-dependent manner [82]. This synergistic effect is mediated by Protein Kinase C (PKC) activation. Furthermore, EGF mediates osteogenic differentiation of dental pulp mesenchymal stem cells [83]. *In vivo*, EGF displayed a synergistic effect with human recombined BMP-2 (hrBMP-2) on bone formation in rat calvarial defects and in tibial defects of rabbits [84,85].

EGF plays a role in signalling in osteoclastogenesis. EGF and TGF-α enhance osteoclast formation in cultures of human marrow, and they promote bone resorption in organ cultures of rat long bones and calvarial bones [86,87]. Moreover, osteoclast proliferation is regulated by EGF-dependent regulation of Osteoprotegerin (OPG) and monocyte chemoattractant protein 1 (MCP1) expression in osteoblasts [88]. Overall, these studies demonstrate a clear involvement of the EGF pathway in bone metabolism. However, EGF can regulate bone metabolism positively or negatively.

This apparently contradictory effect of the EGF signalling pathway on osteogenesis may be explained by the different experimental conditions, such as the combination of several growth factors or different cell cultures, but also by the different EGF concentrations and temporal distribution. Indeed, strong and continuous signalling from EGF promotes MSC osteogenic differentiation, whereas weak and alternating signalling inhibits differentiation [78,89,90].

#### Overview

EGF and EGF-like proteins clearly promote cell proliferation of osteoblasts and osteoclasts. However, the function of EGF signalling pathway in osteoblast differentiation is still controversial given the differences in the experimental settings. It is highly possible that EGF can differently modulate osteogenesis *in vivo* depending on the environmental background.

### Role of EGF signalling pathway in endochondral bone development

EGF signalling is also greatly implicated in postnatal growth [91]. This pathway regulates mammary gland development and lactation, thus favouring postnatal maturity [24]. Moreover, EGF-R knockout mice die before birth or they present different degrees of growth retardation. Over the years, EGF signalling in limb development has been extensively studied. Cell culture of avian growth plate chondrocytes showed that EGF in combination with Growth Hormone (GH) significantly increase proliferation [92].

Immunohistochemistry and *in situ* hybridisation analyses have shown that TGF-α and TGF-α mRNA are uniformly distributed in the limb-forming mesoderm of embryonic chicks at very early stages of differentiation, whereas they are almost absent in non-limb forming areas [93]. At later stages, neither TGF-α nor EGF are present in cartilage or muscle. Moreover, in limb-bud explants *in vitro* cultures, exogenous TGF-α or EGF dramatically increase proliferation but inhibit *in vitro* chondrogenesis. Micromass *in vitro* cultures of mesenchymal cells confirmed these findings, as TGF-α or EGF addition prevents chondrocytes differentiation [93]. In particular, the addition of TGF-α to micro masses of rat limb buds decreases cartilage nodule formation up to 50% in a dose-dependent manner [94]. EGF signalling pathway acts on mesenchymal cells by downregulating PKCα, which in turn leads to ERK-1 phosphorylation, and ultimately to inhibition of chondrogenesis [95]. EGF also performs its action by inhibiting the p38 MAP kinase. Therefore, EGF plays an important role in preventing pre-cartilage condensation. *In vitro* EGF interacts with IGF-1, an important growth factor in skeletal development, by increasing IGF-1 receptors’ number and responsiveness to signals [96].

Interestingly, BTC promotes the proliferation of mesenchymal stem cells and pre-osteoblasts but it inhibits differentiation. This effect is mediated by Hypoxia Inducible Factor 1 alpha (HIF-1α), which is essential for endochondral bone development [10,11,97]. These findings further prove that endochondral ossification is finely tuned by the EGF signalling pathway.

More recently, *in vivo* models have been analysed to confirm the essential role of the EGF pathway in bone growth. EGFR knockout mice were stillbirth or lived only up to 6–8 days, displaying epithelial alterations and dysfunctions in several tissues, such as lungs and intestine. Interestingly, up to one-third of those mice had cleft palates, from retardation in skeletal development [98,99]. In humans, the cleft palate has been associated with polymorphism in TGFA gene, encoding for TGF-α, thus linking this condition to the EGF pathway [100]. Palate growth requires a correct development of the mandibular Meckel’s cartilage, and palate explants of *Egfr^-\-^* mice showed a decrease in the dimensions of Meckel’s cartilage, with the presence of undifferentiated cells with lower content of proteoglycans, consistent with an EGF-dependent modulation of chondrogenesis [101]. MMPs, downstream targets of EGF, mediate this phenotype [101]. Moreover, *in vitro* culture of palate organoids with human MSCs from the umbilical cord demonstrated that EGF significantly promoted proliferation, further proving its involvement in osteogenesis [102]. EGF-like protein signalling has also been associated with other human conditions characterised by growth retardation and skeletal abnormalities from impaired endochondral ossification [103,104].

To better understand the role of EGF signalling in postnatal growth, a rescue experiment was performed [105,106]. In this study, a conditional knock-in mouse model for human EGFR was bred with *Egfr^-\-^* mice to analyse the effects of lower levels of EGFR in different tissues, as the rescue was only partial. In long developing bones, the hypertrophic chondrocyte layer was expanded, probably from a delay in the formation of the primary ossification centre. Moreover, the culture of primary osteoblasts was characterised by decreased proliferation; conversely, differentiation was promoted [105,106].

Further *in vivo* studies have been performed to better clarify whether postnatal growth delay was EGF-dependent. Transgenic mice overexpressing human EGF were characterised by a significant decrease in body weight at birth and by alterations in endochondral development [107]. Namely, hEGF was detected in some proliferating and in all hypertrophic chondrocytes and its expression lead to a delay of hypertrophy. Moreover, an abnormal accumulation of osteoblasts was found both at the periosteum and at the endosteum of long bones, associated with a decrease in cortical bone thickness [107].

As mentioned above, the EGFR signalling is involved both in bone deposition and resorption [108,109]. Indeed, EGFR mRNA was detected *in vitro* in osteoclasts. Moreover, a significantly decreased number of TRAP + cells, namely pre-osteoclasts, were detected in *Egfr^-\-^* mice at E16.5; these cells in wild type mice were mainly distributed inside the calcifying cartilage, whereas in *Egfr^-\-^* mice these cells lie at the periphery of the hypertrophic layer. This phenotype was then rescued at E18.5 and at birth. E18.5 *Egfr-\-* mice were also characterised both by an enlargement of the hypertrophic layer and by the presence of very few bone trabeculae, probably from a delay in the initial recruitment of osteoblasts, as indicated by the altered distribution of these cells in the primary ossification centre [108]. Also, trabecular bone mass was reduced up to birth in these knockout mice, possibly from impaired cell proliferation [49,50].

Modulation of EGF-like proteins produces similar phenotypes. TGF-α newborn knockout mice displayed shorter limbs, enlarged hypertrophic layer, and delayed secondary ossification; interestingly, the phenotype was rescued after 10 weeks [110].

Transgenic mice overexpressing BTC displayed growth retardation and reduced bone dimensions [111]. Histomorphometric analyses of transgenic bones showed a significant increase in bone mineral density (BMD) of femora but not in vertebrae, probably from a different spatial expression of the transgene. Moreover, cortical thickness was significantly higher in those mice compared to controls, and the number and thickness of trabeculae were also increased; lastly, the hypertrophic layer of the growth plate was reduced. These phenotypes were EGFR-dependent, consistently with previous findings [111,112].

However, whole knockout models do not allow for a clear definition of the role of a molecule or a pathway in a specific tissue; thus, conditional knockouts have been recently developed to overcome this issue. Related to the EGF signalling pathway, a limb bud specific knockout mouse model has been analysed; in these animals, a negative regulator of ErbB2 (Herstatin) has been activated under the control of Prx1 promoter, which is expressed in the limb bud mesenchyme. Embryos displayed several alterations in endochondral development: shorter limbs, an enlarged hypertrophic layer, and a delayed primary ossification centre. Moreover, chondrocyte proliferation was impaired and expression of osteocalcin in primary osteoblasts was reduced. Interestingly, the phenotype was completely rescued by day 18.5 [113]. Conditional knockout of ADAM17, an essential disintegrin for the EGF signalling pathway, and of its regulators (Rhomboids 1 and 2) in chondrocytes confirmed these findings, as transgenic mice displayed a significant expansion of the hypertrophic layer, probably from an alteration in bone remodelling [114–116].

Moreover, conditional knockout of EGFR in preosteoblasts and osteoblasts caused shorter limbs in adult mice, and histomorphometric analyses confirmed a striking bone phenotype characterised by reduced trabecular bone volume from reduced trabecular number and thickness, and reduced cortical area, thus proving an alteration in bone formation [117]. These findings were also confirmed by the significant reduction in mesenchymal cell and osteoblast number [117].

In the end, the inhibition of EGF signalling affects several stages of endochondral bone development. This pathway positively regulates chondrocyte proliferation, negatively modulates hypertrophy and favours cartilage replacement by bone. Also, overexpression of HB-EGF in osteoprogenitor cells leads to postnatal chondrodysplasia, chondromas and shorter long bones from increased cell proliferation associated with inhibition of osteogenic differentiation [118].

Overall, these *in vitro* and *in vivo* results suggest that EGF signalling is essential in the very early stages of limb development by maintaining cells in an undifferentiated stage; thus, the decrease of EGF expression is critically important to promote the first phases of chondrogenesis.

#### Overview

EGF and EGF-like proteins are expressed in the limb bud mesenchyme. This pathway contributes to mesenchymal cell proliferation, thus favouring the first proliferative phase of endochondral bone development. Negative modulation of its expression is then critical to support the next phases of endochondral ossification.

### Future and outlook

This comprehensive review of the literature highlights the function of EGF in bone development. This pathway is clearly involved in the first phases of endochondral ossification and in bone homeostasis. However, some points are still controversial and should be further investigated. A better knowledge of the *in vivo* modulation of this pathway in osteogenesis is desirable. As reported above, the spatial and temporal distribution of EGF activity is critical to determine the final effect on bone cells. Moreover, a deeper understanding of the role of EGF in the fine balance between bone formation and bone resorption would allow to plan novel therapeutic approaches to bone pathologies.

## Discussion

EGF and EGF-like proteins finely tune the proliferation and differentiation of bone tissue cells, and they also regulate the initial phases of endochondral ossification. These findings are crucial in light of possible clinical applications. Indeed, several orthopaedic conditions may benefit from the activation of the EGF signalling pathway. For example, EGF or EGF-like proteins together with other stimuli (i.e. BMP-2) may favour the first phases of fracture healing, which recapitulate endochondral bone development [84,85]. In particular, the use of AREG in combination with scaffolds may stimulate MSC migration, thus favouring osteogenesis in the early phases of nonunions [67].

Thus, the EGF signalling pathway may enhance bone restoration in nonunions, characterised by a delay in fracture healing from poor vascularisation or from a lack of mechanical stability at the site of fracture [119,120]. Moreover, EGF may be combined with osteogenic scaffolds to fill large bone losses caused by traumas or tumours [47,121–124].

Further *in vitro* and *in vivo* investigations are necessary to better define the ideal conditions for bone restoration. As mentioned above, different experimental conditions, such as spatial and temporal distribution of the molecule or cell type, can lead to seemingly contrasting results, with EGF promoting or inhibiting cell proliferation and differentiation.

Furthermore, the EGF pathway is clearly involved in several malignancies [26]; thus, preclinical studies *in vitro* and in animal models are required to evaluate the possible long-term oncogenic effects arising from an enhancement of EGF signalling.

## Data Availability

Data sharing is not applicable to this article as no new data were created or analysed in this study.
